# The Frequency and Main Characteristics of Obesity in Undocumented Migrants Receiving Medical Assistance from a Charitable Organisation in Italy

**DOI:** 10.3390/healthcare12232326

**Published:** 2024-11-21

**Authors:** Matteo Franchi, Gianfrancesco Fiorini, Claudia Conflitti, Fabio Riccardo Schibuola, Antonello Emilio Rigamonti, Alessandro Sartorio, Giovanni Corrao, Silvano Gabriele Cella

**Affiliations:** 1 National Centre for Healthcare Research and Pharmacoepidemiology, University of Milano-Bicocca, 20126 Milano, Italy; claudia.conflitti@unimib.it; 2 Section of Biostatistics, Epidemiology and Public Health, Department of Statistics and Quantitative Methods, University of Milano-Bicocca, 20126 Milan, Italy; 3 Istituti Clinici Zucchi, Gruppo San Donato, 20052 Monza, Italy; gianfrancesco.fiorini@grupposandonato.it; 4 Laboratory of Clinical Pharmacology and Pharmacoepidemiology, Department of Clinical Sciences and Community Health, University of Milano, 20122 Milano, Italy; antonello.rigamonti@unimi.it (A.E.R.); silvano.cella@unimi.it (S.G.C.); 5 Policlinico San Matteo, 27100 Pavia, Italy; fabioriccardo.schibuola@gmail.com; 6 Experimental Laboratory for Auxo-Endocrinological Research, IRCCS Istituto Auxologico Italiano, 28824 Piancavallo-Verbania, Italy; sartorio@auxologico.it; 7 Department of Statistics and Quantitative Methods, University of Milano-Bicocca, 20126 Milano, Italy; giovanni.corrao@unimib.it

**Keywords:** undocumented migrants, obesity, overweight, charitable organisation

## Abstract

Background: Obesity is continually growing not only in medium- and high-income countries but also in low-income countries, from where increasing numbers of migrants arrive in Western countries. We aimed to investigate the frequency and characteristics of obesity in a sample of undocumented migrants, a population for which official health data are not available. Methods: We collected demographic and socio-economic data and information on medical diagnoses and pharmacologic treatments for 341 undocumented migrants consecutively attending the outpatient clinic of a big non-governmental organisation in Milan, Italy, from March to July 2023. To measure obesity, we used both body mass index (BMI) and waist circumference (WC). We used multivariate robust Poisson regression models to calculate prevalence ratios (PRs) and 95% Confidence Intervals (CIs) of overweight or obesity according to socio-demographic conditions and other risk factors. Results: Using BMI, the proportion of migrants with obesity was 28.7% (95% CI 24.0–33.0%) and those with overweight represented 32.3% (95% CI 27.3–37.5%). Obesity was more frequent among Asians (53.9%, 95% CI 37.2–69.9%), followed by Latinos (38.7%, 95% CI 29.6–48.5%) and Eastern Europeans (38.2%, 95% CI 25.4–52.3%). Using WC, 68.3% (95% CI 63.1–73.2%) of migrants had values suggestive of overweight or obesity. In the multivariate analyses, overweight and obesity were more frequent in migrants with older age, with a stable employment, and who had been present in Italy for a long time, as well as in those with CV diseases. Moreover, individuals with obesity needed more medications for the cardiovascular system and for the alimentary tract and metabolism. Conclusions: In our sample of undocumented migrants, overweight and obesity were frequent, representing an important public health issue, considering the difficulty experienced by such individuals in finding access to both prevention and healthcare services.

## 1. Introduction

Obesity is an established cardiovascular (CV) risk factor and is continually growing, with epidemic proportions all over the world [1,2]. Together with obesity, the role of other risk factors such as current smoking, arterial hypertension, diabetes mellitus and psycho-social conditions has long been known as responsible for over 90% of the risk of myocardial infarction and stroke [3,4]. These risk factors are often associated and inter-related, as is the case for obesity and hypertension, diabetes and obesity, and hypertension and diabetes [5,6,7].

A dramatic change has occurred in the epidemiology of CV diseases over the last century. From the mid-20th century, they were the first cause of morbidity and mortality in high-income countries [8], but by the end of the century, they became, and still are, the leading morbidity and mortality cause all over the world; indeed, low-income countries now contribute 80% of global CV mortality [9].

The increase in the prevalence and mortality of CV diseases in lower-income countries is only partially explained by contingent factors such as the number of patients with undiagnosed, untreated, or uncontrolled risk factors, the insufficient levels of prehospital care, and the fact that persons living in lower socio-economic conditions are frequently undertreated [10,11]. To better understand this transition in the epidemiology of CV diseases, it has been suggested to consider another aspect: the fact that they occur in parallel with major changes in social patterns all over the world, including urban transition, nutritional transition, and activity transition [9]. These changes have considerable proportions. For example, in Europe, the proportion of people living in urban contexts has grown from 55% in 1955 to 74% in 2020; while urbanisation is associated with economic growth, it often also entails social deprivation, overcrowding, and greater consumption of foods rich in fats, salt, and sugar instead of vegetables and fresh fruit [12].

In Europe, the impact of socio-economic factors on CV disease epidemiology is evident: the estimated rate of incidence of CV diseases is around 30% higher in middle-income countries than in high-income countries and life expectancy is shorter in the former; at the same time, middle-income countries have atmospheric concentrations of PM_2.5_ twice as high of those in high-income countries and their expenditure for healthcare is only one-quarter of that in high-income countries [12].

Sixty percent of European adults and one-third of children are affected by overweight or obesity, which are more common in persons living in lower socio-economic conditions [13]. This reflects a worldwide trend. Indeed, the prevalence of obesity has increased more rapidly in low- and middle-income countries than in high-income countries due to still unclear gene–environment interactions [14]. It can therefore be expected that people migrating to Europe from other parts of the world share, maybe even to a larger extent, problems of obesity with all its consequences. This is going to have an increasing impact on the health systems of the hosting countries, given the continuous increase in the number of international migrants which in 2020 reached 281 million. Among them, the proportion of those aged 65 and above is also increasing; in mid-2020, they were 34.3 million in number, 12.2% of the total [15]. The problem is made more complicated by the fact that part of these persons are represented by undocumented migrants. An undocumented migrant is “a person who moves or has moved across an international border and is not authorised to enter or to stay in a state pursuant to the law of that state and to international agreements to which that state is a party” [15]. Their number cannot be calculated but it has been estimated to be around 10% of that of all international migrants [16]. These persons can remain in this irregular situation for many years in the host country. For documented migrants, health data can be retrieved from the same data sources containing those of natives. This is not clearly the case with undocumented migrants. Data on their health conditions, and especially on chronic diseases, are scanty, and the available literature is limited and somehow contradictory [17,18,19]. In addition, little information is available on the prevalence of obesity and overweight in these persons, though this seems to be quite high [17] and possibly increases over time once they reside in host countries [2].

The present study aims to describe the frequency of obesity and its main characteristics in a sample of undocumented migrants from different geographical regions receiving health assistance from a big non-governmental organisation (NGO) in Milan, Italy. These persons could have problems of overweight and obesity both because they come from low-income countries and also because, living here in poor socio-economic conditions and possibly on unhealthy diets, they have an increased chance of experiencing such problems. The latter also combines with important practical barriers in accessing healthcare. These include fear of detention, practical obstacles such as transport problems in reaching the clinic [20,21], language, health literacy, cultural beliefs, and a lack of familiarity with the health system [22,23]. Experiencing a barrier to healthcare appears to be more common in persons of younger age, of a lower education level, and with no children [20]. These barriers should always be considered when dealing with undocumented migrants. Especially for chronic conditions, as is the case for obesity, they could be a severe limitation to receiving adequate treatment and also to implementing preventive measures. Recommendations are available to overcome these barriers [24], but they require the involvement of policy-makers and in-depth knowledge of the health problems of these patients. Our study is a contribution to this topic.

## 2. Materials and Methods

The target population includes all of the undocumented migrants consecutively attending the outpatient clinic of Opera San Francesco between 1 March 2023 and 31 July 2023. Opera San Francesco is a big NGO, based in Milan, Italy, that gives medical assistance for free to the poor. Doctors work there on a voluntary basis, and they offer both working day general practice and consultations in all specialties. People coming as outpatients are first seen by a general practitioner who can refer them for further investigations or to a specialist. The general practitioner also prescribes medications which are then dispensed for free by the pharmacists of the NGO. Medical notes and dispensed medicines are recorded in an electronic dataset, which is updated every time the individual comes to be seen. In this way, persons are completely free to come for every health necessity. At the same time, their medical history is always available, through structured electronic databases, for all professionals entitled to access it. Each individual is asked to sign an informed consent form before being enrolled, which is available in the languages most commonly spoken by this particular population, among which are Italian, English, French, and Arabic.

We collected demographic and socio-economic data and information on medical diagnoses and pharmacologic treatments. In particular, the following information was retrieved: geographic area of origin, sex, age, years spent in Italy, level of education, type of employment (unemployed, precarious or occasional or stable), availability of a stable accommodation, and regular use of alcohol and/or cigarettes. This was done first with an interview, using a written questionnaire translated into all of the aforementioned languages, and then by seeking the records of the individual in the study database. Persons younger than 18 years, pregnant women, and women with post-partum lactation were excluded from the study. We measured weight, height, and waist circumference at the navel in a standing position, after having breathed out naturally [25]. Body mass index (BMI) and waist circumference were used to evaluate the presence of obesity. Specifically, the following cut-offs were used: underweight (BMI ≤ 18.5); normal weight (18.5 < BMI ≤ 24.9); overweight (24.9 < BMI ≤ 29.9); obese class I (29.9 < BMI ≤ 35.9); obese class II (35.9 < BMI ≤ 39.9); obese class III (BMI > 39.9). For Asians, the following cut-offs were used instead: underweight (BMI < 18.5); normal weight (18.5 ≤ BMI ≤ 22.9); overweight (23 ≤ BMI ≤ 24.9); obese class I (24.9 < BMI ≤ 29.9); obese class II (BMI > 29.9) [26].

Given the small number of subjects in some ethnic groups, when using BMI, we considered individuals with obesity and overweight together, i.e., all those with a BMI value ≥ 25 kg/m^2^, or a BMI value ≥ 22.9 for Asians. Using waist circumference, values > 80 cm in women and >94 cm in men were associated with a significantly higher risk of CV disease and both overall and cause-specific mortality [27]. The corresponding figures for Asian patients were >80 cm in women and >90 cm in men [28]. We did not use the recently proposed waist-to-height ratio, since the recommendation of keeping “the size of your waist to less than half of your height” penalises shorter people [29]. To all the participants in the study, we administered a validated questionnaire measuring the level of compliance with the Mediterranean diet [30]. Diabetes and CV diseases were considered to be present in persons consuming regular anti-diabetic and CV medications as defined on the basis of the Anatomical Therapeutic and Chemical (ATC) Classification, as previously described [31].

### 2.1. Statistical Analysis

Considering an expected prevalence of obesity of 12% as in the general Italian population [32] and a first type error (α) of 0.05, 332 individuals would have allowed us to estimate a prevalence of obesity with an absolute error of 3.5%.

Baseline characteristics were expressed as absolute and percentage frequencies. Differences in the frequencies of continuous variables were assessed by using the t-test, the ANOVA test, or their non-parametric versions. Differences among categorial variables were assessed by using the χ^2^ test, or its version for trend or the Fisher’s exact test, as appropriate. The percentage of individuals with overweight/obesity were reported along with 95% Confidence Intervals (CI). Multivariable robust error variance Poisson regression models were used to measure prevalence ratios (PRs) and the corresponding 95% CIs of overweight and obesity according to the baseline characteristics. Given the high percentage of missing data for the number of cohabitants, this variable was not included in the model. However, a sensitivity analysis was performed in order to deal with missing data, using a multiple imputation technique based on the fully conditional specification (FCS) method [33]. Statistical Analysis System software (V.9.4; SAS Institute, Cary, NC, USA) was used for the statistical analysis. For all hypotheses tested, two-tailed *p* values < 0.05 were considered significant.

### 2.2. Ethics Approval

This study was approved by the Ethics Committee of the University of Milano-Bicocca (Prot. N. 0179418, 5 May 2023). Each participant signed an informed consent form before entering the study. The data were completely and permanently anonymised.

## 3. Results

Overall, 347 individuals were asked to sign the informed consent form to participate in the study. Six individuals (five from North Africa and one from Eastern Europe) opted not to participate; thus, they did not sign the informed consent form and were therefore excluded from the study. The flowchart of the selection of the study population is shown in Supplementary Figure S1. Table 1 shows the main characteristics of the 341 individuals included in the study. Among these, 87 were from North Africa, 49 were from South Africa, 111 were from Latin America, 39 were from Asia, and 55 from were Eastern Europe. No persons from the Middle East sought assistance from the NGO during the period of the study. There were 184 (54.0%) males, and the mean age was 41.3 ± 12.2 years. Latin Americans were the most represented ethnic group; they were also the group with the highest percentage of females. Differences were observed in the age distribution, with South Africans being the youngest population (average age: 37 years) and Europeans the oldest (average age: 46 years). Latin Americans had the shortest length of stay in Italy, while Eastern Europeans were those with the longest. Smoke, either alone or in combination with alcohol intake, was most frequent in Eastern Europeans, while smoke alone was most frequent in North Africans. Asians and Latin Americans were those who most frequently had a job and stable housing.

**Figure 1:**
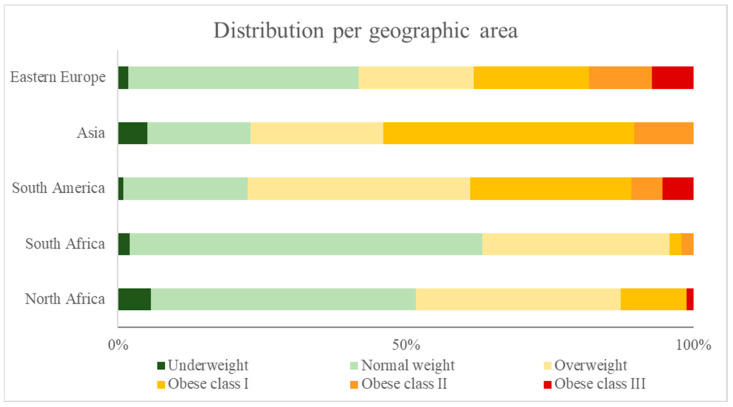
Weight distribution of cohort, stratified by geographic area of origin and based on BMI values.

Overall, using BMI, individuals with obesity represented 28.7% (95% CI 24.0–33.9%) and those with overweight represented 32.3% (95% CI 27.3–37.5%), but there were significant differences among different ethnic groups (*p* < 0.001 for both obesity and overweight), with Latinos being those with the highest frequency of overweight/obesity (77.5%, 95% CI 68.6–84.9%), closely followed by Asians (76.9%, 95% CI 60.7–88.9%) (Figure 1). Similar results were observed when obesity was diagnosed according to waist circumference, with an overall frequency of overweight/obesity of 68.3% (63.1–73.2%) (Table 2).

In the multivariable analysis, the frequency of obesity or overweight was significantly different according to ethnic groups, and it was higher among migrant females, those with older age, a low number of years spent in Italy, and stable employment, and in those suffering from hypertension. No significant differences were observed according to level of education, having accommodation, adherence to the Mediterranean diet, regular use of alcohol and/or cigarettes, and diabetes (Table 2). The results based on the multiple imputation of missing data are reported in the Supplementary Materials (Table S1).

When we looked at the use of medications, we noticed some differences between persons with and without obesity (or overweight). Individuals with obesity used more drugs for the alimentary tract and metabolism (which include antidiabetic agents) and for the CV system (Table 3).

## 4. Discussion

Obesity has reached pandemic proportions. Besides being a well-known CV risk factor, its possible role in the etiopathogenesis of a variety of chronic diseases is increasingly reported [34,35,36,37,38]. However, very little data are available on its prevalence in undocumented migrants, who have become a stable component of the population in Western countries.

In this study we observed that undocumented migrants are frequently affected by overweight or obesity, as measured either with BMI or WC. These two methods seemed to give comparable results in the population of this study, and obesity appeared to be unequally distributed among persons coming from different geographical areas, with the highest frequency in Asians and Latin Americans.

Individuals with obesity were older, were in Italy for a shorter period, and more frequently had stable employment. Females appear to be more involved than males. Also, in our population, obesity was more frequent in persons with hypertension.

The observation that the Asian group had a high frequency of obesity seems important since this ethnic group has a high risk of diabetes and cardiovascular diseases [39]. Moreover, it is known that South Asians are at an increased risk of developing diabetes and CV diseases at a lower BMI, compared to other ethnic groups [40].

The fact that Latinos are also frequently affected by obesity is in keeping with data obtained in the US, where they are a significant part of the population [41]. Though they are usually considered one ethnic group, they are a very heterogenous mix of genetic ancestry, culture, and environmental conditioning. In our population, they are mainly represented by South Americans, while in the US, Mexicans comprise a great proportion. In spite of this difference, obesity appears to affect both of these Latino populations. Indeed, it has been demonstrated that while obesity prevalence in Latinos differs by country of origin, it is high in all of them, but South Americans seem to be at a lower risk than other Latino groups [42,43].

Although the length of stay in the host country has been demonstrated to be associated with a greater prevalence of obesity and other CV risk factors [44,45,46], the importance of this fact could now be decreasing in consideration of the worsening obesity epidemics affecting low- and middle-income countries [14]. Our groups with a high frequency of obesity had different lengths of stay in Italy: the shortest for Latinos and intermediate for Asians.

Surely many other factors are involved in the development of obesity in addition to length of stay in the host country, ethnicity, and geographical area of origin. Among these, genome-wide association studies and epigenome-wide association studies could prove of crucial importance in the coming years, provided that attention is given to the correct enrolment of individuals. So far, for example, studies carried out in the US have involved mostly individuals of European ancestry, while the majority of subjects with obesity are of Hispanic Latino and African American descent [47].

Overall, obesity was more frequent in females and those of older age; its frequency did not substantially vary among different degrees of education. It was more frequent in those currently employed and with stable housing, independent of the number of housemates. Some of these considerations are in agreement with observations of other researchers, for example the higher frequency of obesity in females than in males [48,49]. Other aspects are difficult to explain in light of what has been widely reported. For example, it has been observed that obesity is more frequent in persons with lower socio-economic status [49,50,51], but in our study, we cannot draw any firm conclusion on this point.

Quite expectedly, also in our group of migrants, hypertension was more frequent among patients with obesity, as already known [5,52]. This was also true for diabetes, though in this case our results did not reach statistical significance.

Finally, we observed that individuals with obesity were more frequently on specific medications, i.e., those labelled in the ATC system as drugs for CV system and for alimentary tract and metabolism, the latter comprising all antidiabetic agents. This gives further relevance to the above observation; also, in our population individuals with obesity needed more treatment for CV problems and diabetes.

Another ATC class of medication more often used by patients with obesity was that of drugs for the nervous system, which includes painkillers such as paracetamol. This appears to be of interest in light of a recent study showing that chronic pain is more frequent and more severe in persons who have experienced migration; in this population, many factors are associated with chronic pain and include female gender and weight alterations (underweight and obesity) [53].

Our study has some limitations, the main being the low number of individuals in some subgroups. This in turn made it impossible to better define the characteristics of migrants on the basis of their geographical origin. This appears to be especially important for adherence to a healthy diet, in consideration of the fact that this is one of the major modifiable factors when implementing interventions to reduce obesity. However, migrants may prefer to eat their traditional diet, instead of adhering to the Mediterranean diet, and this may explain the lack of correlation with obesity. Moreover, it should be emphasised that our study sample included undocumented migrants who seek health assistance from Opera San Francesco and is thus not representative of the overall undocumented migrant population. For this reason, our results cannot be generalised to the broader population of migrants. However, we believe that these data are extremely important for underlining that overweight and obesity represent an important public health issue in this particular population. Moreover, given the lack of such data in the current literature, our study may be helpful to increase the knowledge about this issue in the undocumented migrant population.

## 5. Conclusions

Obesity and overweight are frequent in our sample of undocumented migrants, and this represents an important public health issue, especially considering the difficulty experienced by such individuals in finding access to both prevention and healthcare services.

## Figures and Tables

**Table 1:** Characteristics of total cohort and stratified by geographic area of origin.

	Total	North Africa	South Africa	Latin America	Asia	Eastern Europe	*p*-Value
	N, %	N, %	N, %	N, %	N, %	N, %	
N	341	87	49	111	39	55	
Sex							
Male	184, 54.0	75, 86.2	41, 83.7	20, 18.0	26, 66.7	22, 40.0	<0.001
Female	157, 46.0	12, 13.8	8, 16.3	91, 82.0	13, 33.3	33, 60.0	
Age (mean (SD))	41.30 (12.2)	36.97 (12.2)	38.53 (11.7)	43.51 (11.7)	41.39 (10.2)	46.07 (12.3)	
18–30	79, 23.2	35, 40.2	16, 32.7	16, 14.4	4, 10.3	8, 14.5	<0.001
31–40	74, 21.7	15, 17.3	9, 18.3	28, 25.3	15, 38.4	7, 12.7	
41–50	93, 27.3	19, 21.8	16, 32.7	33, 29.7	11, 28.2	14, 25.5	
51+	95, 27.8	18, 20.7	8, 16.3	34, 30.6	9, 23.1	26, 47.3	
Years spent in Italy (mean (SD))	7.33 (8.9)	9.30 (11.0)	7.61 (8.6)	3.80 (5.3)	6.80 (8.6)	11.4 (8.9)	
Education							
Illiterate	21, 6.2	4, 4.6	9, 18.4	0	1, 2.6	7, 12.7	<0.001
Elementary school	51, 14.9	8, 9.2	16, 32.6	12, 10.8	7, 17.9	8, 14.6	
Middle school	120, 35.2	37, 42.5	8, 16.3	43, 38.8	15, 38.5	17, 30.9	
High school	102, 29.9	21, 24.2	7, 14.3	49, 44.1	8, 20.5	17, 30.9	
University	46, 13.5	17, 19.5	9, 18.4	7, 6.3	8, 20.5	5, 9.1	
Missing	1, 0.3					1, 1.8	
Employment							
Unemployed	184, 54.0	50, 57.5	37, 75.5	45, 40.6	18, 46.2	34, 61.8	<0.001
Precarious/occasional	39, 11.4	7, 8.0	2, 4.1	20, 18.0	2, 5.1	8, 14.6	
Stable	117, 34.3	30, 34.5	9, 18.4	46, 41.4	19, 48.7	13, 23.6	
Missing	1, 0.3		1, 2.0				
Housing							
Stable housing	283, 83.0	65, 74.7	27, 55.1	109, 98.2	35, 89.7	47, 85.4	<0.001
Homeless	58, 17.0	22, 26.3	22, 44.9	2, 1.8	4, 10.3	8, 14.6	
Cohabitants (mean (SD))	3.33 (1.7)	3.50 (1.8)	3.46 (2.1)	3.20 (1.6)	3.63 (1.6)	3.06 (1.6)	<0.001
Missing	57, 16.7	21, 24.1	21, 42.9	3, 2.7	4, 10.3	8, 14.5	
Adherence to Mediterranean diet							
Low	197, 57.8	37, 42.5	29, 59.2	75, 67.6	16, 41.0	40, 72.7	<0.001
Medium	96, 28.1	31, 35.6	15, 30.6	30, 27.0	10, 25.7	10, 18.2	
High	48, 14.1	19, 21.9	5, 10.2	6, 5.4	13, 33.3	5, 9.1	
Substance use							
No	229, 67.2	47, 54.0	39, 79.6	87, 78.4	33, 84.6	23, 41.8	
Alcohol	19, 5.6	1, 1.2	4, 8.2	13, 11.7	0	1, 1.8	<0.001
Cigarettes	76, 22.3	35, 40.2	6, 12.2	8, 7.2	6, 15.4	21, 38.2	
Cigarettes and alcohol	17, 5.0	4, 4.6	0	3, 2.7	0	10, 18.2	
Diabetes	35, 10.3	10, 11.5	3, 6.1	11, 9.9	6, 15.4	5, 9.1	0.688
CV diseases	55, 16.1	13, 14.9	6, 12.2	19, 17.1	5, 12.8	12, 21.8	0.672

**Table 2:** Prevalence ratio (PR) of being obese or overweight according to individual characteristics. Definition of obesity and overweight based on (a) BMI; (b) waist circumference (WC).

Total	N	Obese or Overweight BMI	PR (95% CI)	Obese or Overweight WC	PR (95% CI)
	341	208 (61.0%)		233 (68.3%)	
Geographic area					
North Africa	87	42 (48.3%)	0.76 (0.59–0.98)	52 (59.7%)	0.94 (0.75–1.16)
South Africa	49	18 (36.7%)	0.57 (0.37–0.86)	18 (36.7%)	0.62 (0.41–0.91)
Latin America	111	86 (77.5%)	Ref.	98 (88.3%)	Ref.
Asia	39	30 (76.9%)	0.98 (0.78–1.23)	27 (69.2%)	0.93 (0.73–1.16)
Eastern Europe	55	32 (58.2%)	0.79 (0.62–1.02)	38 (69.1%)	0.92 (0.74–1.14)
Sex					
Male	184	101 (54.9%)	Ref.	100 (54.4%)	Ref.
Female	157	107 (68.2%)	0.95 (0.80–1.14)	133 (84.7%)	1.35 (1.14–1.62)
Age					
18–30	79	25 (31.7%)	Ref.	43 (54.4%)	Ref.
31–40	74	45 (60.8%)	1.66 (1.15–2.38)	48 (64.9%)	1.10 (0.86–1.40)
41–50	93	69 (74.2%)	2.09 (1.50–2.93)	69 (74.2%)	1.38 (1.10–1.73)
51+	95	66 (72.6%)	1.95 (1.37–2.75)	73 (76.8%)	1.37 (1.09–1.72)
Years spent in Italy					
≤1	126	75 (59.5)	Ref.	96 (76.2)	Ref.
>1	215	133 (61.9)	0.90 (0.76–1.07)	137 (63.7)	0.76 (0.66–0.89)
Education *					
Illiterate	22	14 (63.4)	1.22 (0.85–1.75)	15 (68.2)	1.13 (0.86–1.49)
Elementary school	51	28 (54.9)	0.92 (0.71–1.19)	30 (58.8)	0.88 (0.71–1.10)
Middle school	120	75 (62.5)	Ref.	83 (69.2)	Ref.
High school/university	148	91 (61.5)	1.00 (0.85–1.19)	105 (71.0)	1.00 (0.87–1.17)
Employment *					
Unemployed/precarious/occasional	223	125 (56.1)	Ref.	140 (62.8)	Ref.
Stable	117	83 (70.9)	1.11 (0.95–1.31)	93 (79.5)	1.18 (1.04–1.35)
Housing					
Homeless	58	22 (37.9)	Ref.	26 (44.8)	Ref.
Stable housing	283	186 (65.7)	1.28 (0.95–1.73)	207 (73.1)	1.10 (0.83–1.47)
Adherence to Mediterranean diet					
Low/medium	199	177 (60.4)	Ref.	199 (67.9)	Ref.
High	34	31 (64.6)	1.06 (0.84–1.34)	34 (70.8)	1.11 (0.90–1.37)
Substance use					
No	229	149 (65.1)	Ref.	162 (70.7)	Ref.
Alcohol and/or cigarettes	112	59 (52.7)	0.91 (0.76–1.10)	71 (63.4)	1.03 (0.87–1.20)
Diabetes					
No	306	179 (58.5)	Ref.	205 (67.0)	Ref.
Yes	35	29 (82.9)	1.06 (0.85–1.30)	28 (80.0)	1.03 (0.84–1.25)
Hypertension					
No	286	161 (56.3)	Ref.	187 (65.4)	Ref.
Yes	55	47 (85.5)	1.31 (1.09–1.59)	46 (83.6)	1.23 (1.03–1.47)

* One missing value was excluded from this analysis. PR: prevalence ratio.

**Table 3:** Use of medications (at least one prescription) in individuals with and without obesity (and overweight).

	Obese or Overweight * (*n* = 208)	Not Obese or Overweight * (*n* = 133)	
ATC Class of Drugs	N, %	N, %	*p*-Value ^α^
Alimentary tract and metabolism	84, 40.4%	34, 25.6%	0.0050
Blood and blood-forming organs	22, 10.6%	14, 10.5%	0.9882
Cardiovascular system	47, 22.6%	8, 6.0%	<0.0001
Dermatological drugs	22, 10.6%	11, 8.3%	0.4823
Genitourinary system and reproductive hormones	9, 4.3%	10, 7.5%	0.2101
Systemic hormonal preparations, excluding reproductive hormones and insulins	20, 9.6%	7, 5.3%	0.1466
Anti-infectives for systemic use	40, 19.2%	26, 19.6%	0.9422
Antineoplastic and immunomodulating agents	1, 0.5%	1, 0.8%	1.0000
Musculoskeletal system	79, 38.0%	41, 30.8%	0.1773
Nervous system	58, 28.0%	27, 20.3%	0.1143
Antiparasitic products, insecticides, and repellents	3, 1.4%	0	0.2842
Respiratory system	38, 18.3%	18, 13.5%	0.2496
Sensory organs	21, 10.1%	9, 6.8%	0.2898
Various ATC structures	1, 0.5%	1, 0.8%	1.0000
Vitamin C	1, 0.5%	0	1.0000
Does not take any medication	35, 16.8%	32, 24.1%	0.1011
Missing data	16, 7.7%	6, 4.5%	0.2435

* Defined by using body mass index; ^α^ *p*-value of Chi-square test or Fisher’s exact test.

## Data Availability

The data presented in this study are available on request from the corresponding author.

## Supplementary Materials

The following supporting information can be downloaded at: https://www.mdpi.com/article/10.3390/healthcare12232326/s1, Figure S1: Flowchart of study population selection; Table S1: Prevalence ratio (PR) of being obese or overweight according to individual characteristics. Definition of obesity and overweight based on body mass index. Multiple imputation was adopted to account for missing data.

## References

[1] Hajar R. (2016). Framingham Contribution to Cardiovascular Disease. Heart Views.

[2] NCD Risk Factor Collaboration (NCD-RisC) (2016). Trends in adult body-mass index in 200 countries from 1975 to 2014: A pooled analysis of 1698 population-based measurement studies with 19·2 million participants. Lancet.

[3] Yusuf S., Hawken S., Ôunpuu S., Dans T., Avezum A., Lanas F., McQueen M., Budaj A., Pais P., Varigos J. (2004). INTERHEART Study Investigators. Effect of potentially modifiable risk factors associated with myocardial infarction in 52 countries (the INTERHEART study): Case-control study. Lancet.

[4] O’Donnell M.J., Chin S.L., Rangarajan S., Xavier D., Liu L., Zhang H., Rao-Melacini P., Zhang X., Pais P., Agapay S. (2016). INTERSTROKE investigators. Global and regional effects of potentially modifiable risk factors associated with acute stroke in 32 countries (INTERSTROKE): A case-control study. Lancet.

[5] Susic D., Varagic J. (2017). Obesity: A Perspective from Hypertension. Med. Clin. N. Am..

[6] Malone J.I., Hansen B.C. (2019). Does obesity cause type 2 diabetes mellitus (T2DM)? Or is it the opposite?. Pediatr. Diabetes.

[7] Wu Y., Hu H., Cai J., Chen R., Zuo X., Cheng H., Yan D. (2021). Association of hypertension and incident diabetes in Chinese adults: A retrospective cohort study using propensity-score matching. BMC Endocr. Disord..

[8] GBD 2015 Mortality and Causes of Death Collaborators (2016). Global, regional, and national life expectancy, all-cause mortality, and cause-specific mortality for 249 causes of death, 1980–2015: A systematic analysis for the Global Burden of Disease Study 2015. Lancet.

[9] Teo K.K., Rafiq T. (2021). Cardiovascular Risk Factors and Prevention: A Perspective From Developing Countries. Can. J. Cardiol..

[10] Prabhakaran D., Jeemon P., Roy A. (2016). Cardiovascular Diseases in India: Current Epidemiology and Future Directions. Circulation.

[11] Zhao D., Liu J., Wang M., Zhang X., Zhou M. (2019). Epidemiology of cardiovascular disease in China: Current features and implications. Nat. Rev. Cardiol..

[12] Timmis A., Vardas P., Townsend N., Torbica A., Katus H., De Smedt D., Gale C.P., Maggioni A.P., Petersen S.E., Huculeci R. (2022). European Society of Cardiology. European Society of Cardiology: Cardiovascular disease statistics 2021. Eur. Heart J..

[13] Vandevijvere S., De Pauw R., Djojosoeparto S., Gorasso V., Guariguata L., Løvhaug A.L., Mialon M., Van Dam I., von Philipsborn P. (2023). Upstream Determinants of Overweight and Obesity in Europe. Curr. Obes. Rep..

[14] Pledger S.L., Ahmadizar F. (2023). Gene-environment interactions and the effect on obesity risk in low and middle-income countries: A scoping review. Front. Endocrinol..

[15] World Health Organization (2022). World Report on the Health of Refugees and Migrants.

[16] (2013). PICUM: PICUM Submission to the UN Committee on the Protection of the Right of All Migrant Workers and Members of their Families. https://www.ohchr.org/sites/default/files/Documents/HRBodies/CMW/Discussions/2013/DGDMigrationData_PICUM_2013.pdf.

[17] Jackson Y., Paignon A., Wolff H., Delicado N. (2018). Health of undocumented migrants in primary care in Switzerland. PLoS ONE.

[18] Gimeno-Feliu L.A., Pastor-Sanz M., Poblador-Plou B., Calderón-Larrañaga A., Díaz E., Prados-Torres A. (2020). Multimorbidity and chronic diseases among undocumented migrants: Evidence to contradict the myths. Int. J. Equity Health.

[19] van de Sande J.S.O., van den Muijsenbergh M.E.T.C. (2017). Undocumented and documented migrants with chronic diseases in Family Practice in the Netherlands. Fam. Pract..

[20] Mona H., Andersson L.M.C., Hjern A., Ascher H. (2021). Barriers to accessing health care among undocumented migrants in Sweden—A principal component analysis. BMC Health Serv. Res..

[21] Fu L., Lindenmeyer A., Phillimore J., Lessard-Phillips L. (2022). Vulnerable migrants’ access to healthcare in the early stages of the COVID-19 pandemic in the UK. Public Health.

[22] Filmer T., Ray R., Glass B.D. (2023). Barriers and facilitators experienced by migrants and refugees when accessing pharmaceutical care: A scoping review. Res. Soc. Adm. Pharm..

[23] Finnigan C., Brown J., Al-Adeimi M., Al-Abed R. (2022). Barriers to Accessing Mental Health Services by Migrant Youth. Community Ment. Health J..

[24] Kanengoni-Nyatara B., Watson K., Galindo C., Charania N.A., Mpofu C., Holroyd E. (2024). Barriers to and Recommendations for Equitable Access to Healthcare for Migrants and Refugees in Aotearoa, New Zealand: An Integrative Review. J. Immigr. Minor. Health.

[25] Obesity: Identification, assessment and management (2023). NICE Guideline, No. 189.

[26] World Health Organization The Asia-Pacific Perspective: Redefining Obesity and Its Treatment. https://iris.who.int/handle/10665/206936.

[27] WHO (2011). Waist Circumference and Waist–Hip Ratio: Report of a WHO Expert Consultation, Geneva, 8–11 December 2008.

[28] Gupta R.D., Parray A.A., Kothadia R.J., Pulock O.S., Pinky S.D., Haider S.S., Akonde M., Haider M.R. (2024). The association between body mass index and abdominal obesity with hypertension among South Asian population: Findings from nationally representative surveys. Clin. Hypertens..

[29] Nevill A.M., Duncan M.J., Myers T. (2022). NICE’s recent guidelines on “the size of your waist” unfairly penalizes shorter people. Obes. Res. Clin. Pract..

[30] Ruggeri S., Buonocore P., Amoriello T. (2022). New Validated Short Questionnaire for the Evaluation of the Adherence of Mediterranean Diet and Nutritional Sustainability in All Adult Population Groups. Nutrients.

[31] Fiorini G., Cerri C., Bini S., Rigamonti A.E., Perlini S., Marazzi N., Sartorio A., Cella S.G. (2016). The burden of chronic noncommunicable diseases in undocumented migrants: A 1-year survey of drugs dispensation by a non-governmental organization in Italy. Public Health.

[32] National Observatory on Health in the Regions. https://osservatoriosullasalute.it/rapporto-osservasalute.

[33] Sterne J.A.C., White I.R., Carlin J.B., Spratt M., Royston P., Kenward M.G., Wood A.M., Carpenter J.R. (2009). Multiple imputation for missing data in epidemiological and clinical research: Potential and pitfalls. BMJ.

[34] Hanusek K., Karczmarski J., Litwiniuk A., Urbańska K., Ambrozkiewicz F., Kwiatkowski A., Martyńska L., Domańska A., Bik W., Paziewska A. (2022). Obesity as a Risk Factor for Breast Cancer-The Role of miRNA. Int. J. Mol. Sci..

[35] Devericks E.N., Carson M.S., McCullough L.E., Coleman M.F., Hursting S.D. (2022). The obesity-breast cancer link: A multidisciplinary perspective. Cancer Metastasis Rev..

[36] Islam M.R., Arthur S., Haynes J., Butts M.R., Nepal N., Sundaram U. (2022). The Role of Gut Microbiota and Metabolites in Obesity-Associated Chronic Gastrointestinal Disorders. Nutrients.

[37] Martínez-Montoro J.I., Morales E., Cornejo-Pareja I., Tinahones F.J., Fernández-García J.C. (2022). Obesity-related glomerulopathy: Current approaches and future perspectives. Obes. Rev..

[38] Jiménez-Cortegana C., Hontecillas-Prieto L., García-Domínguez D.J. (2022). Obesity and Risk for Lymphoma: Possible Role of Leptin. Int. J. Mol. Sci..

[39] Tillin T., Hughes A.D., Mayet J., Whincup P., Sattar N., Forouhi N.G., McKeigue P.M., Chaturvedi N. (2013). The relationship between metabolic risk factors and incident cardiovascular disease in Europeans, South Asians, and African Caribbeans: SABRE (Southall and Brent Revisited)—A prospective population-based study. J. Am. Coll. Cardiol..

[40] Vikram N.K., Pandey R.M., Misra A., Sharma R., Devi J.R., Khanna N. (2003). Non-obese (body mass index < 25 kg/m2) Asian Indians with normal waist circumference have high cardiovascular risk. Nutrition.

[41] Hales C.M., Carroll M.D., Fryar C.D., Ogden C.L. (2017). Prevalence of obesity among adults and youth: United States, 2015–2016. NCHS Data Brief.

[42] Devia C., Flórez K.R., Costa S.A., Huang T.T. (2021). Prevalence of self-reported obesity among diverse Latino adult populations in New York City, 2013-2017. Obes. Sci. Pract..

[43] López-Cevallos D.F., Gonzalez P., Bethel J.W., Castañeda S.F., Isasi C.R., Penedo F.J., Ojeda L., Davis S.M., Chirinos D.A., Molina K.M. (2018). Is there a link between wealth and cardiovascular disease risk factors among Hispanic/Latinos? Results from the HCHS/SOL sociocultural ancillary study. Ethn. Health.

[44] Commodore-Mensah Y., Ukonu N., Obisesan O., Aboagye J.K., Agyemang C., Reilly C.M., Dunbar S.B., Okosun I.S. (2016). Length of Residence in the United States is Associated With a Higher Prevalence of Cardiometabolic Risk Factors in Immigrants: A Contemporary Analysis of the National Health Interview Survey. J. Am. Heart Assoc..

[45] Osibogun O., Ogunmoroti O., Mathews L., Okunrintemi V., Tibuakuu M., Michos E.D. (2021). Greater Acculturation is Associated With Poorer Cardiovascular Health in the Multi-Ethnic Study of Atherosclerosis. J. Am. Heart Assoc..

[46] Anikpo I., Dodds L., Mesa R.A., Tremblay J., Vilchez L., Elfassy T. (2024). Length of Time in the United States and Cardiometabolic Outcomes Among Foreign and US-Born Black Adults. J. Racial Ethn. Health Disparities.

[47] Tirthani E., Said M.S., Rehman A. (2024). Genetics and Obesity. 2023 Jul 31. StatPearls [Internet].

[48] Cooper A.J., Gupta S.R., Moustafa A.F., Chao A.M. (2021). Sex/Gender Differences in Obesity Prevalence, Comorbidities, and Treatment. Curr. Obes. Rep..

[49] Raftopoulou A., Gil Trasfi J. (2024). Income-related inequality in obesity and its determinants in Spain: What happens beyond the obesity threshold?. Int. J. Health Econ. Manag..

[50] Chou S.Y., Grossman M., Saffer H. (2004). An economic analysis of adult obesity: Results from the Behavioral Risk Factor Surveillance System. J. Health Econ..

[51] Esposito L., Villaseñor A., Rodríguez E.C., Millett C. (2020). The economic gradient of obesity in Mexico: Independent predictive roles of absolute and relative wealth by gender. Soc. Sci. Med..

[52] Yamamoto Y., Ikeue K., Kanasaki M., Yamakage H., Satoh-Asahara N., Masuda I., Ishii K. (2024). Age-wise examination of the association of obesity based on body mass index and waist circumference with metabolic diseases in comprehensive health checkup participants. Obes. Sci. Pract..

[53] Tsetseri M.N., Keene D.J., Silman A.J., Dakin S.G. (2024). Exploring the burden, prevalence and associated factors of chronic musculoskeletal pain in migrants from North Africa and Middle East living in Europe: A scoping review. BMC Public Health.

