# Supplementary material for: The Frequency and Main Characteristics of Obesity in Undocumented Migrants Receiving Medical Assistance from a Charitable Organisation in Italy
Source: Healthcare (Basel). 2024 Nov 21;12(23):2326. doi: 10.3390/healthcare12232326 (PMC11641085; doi:10.3390/healthcare12232326)
Supplement: Supplementary file 1 [file healthcare-12-02326-s001.zip › healthcare-3301465-supplementary.pdf]

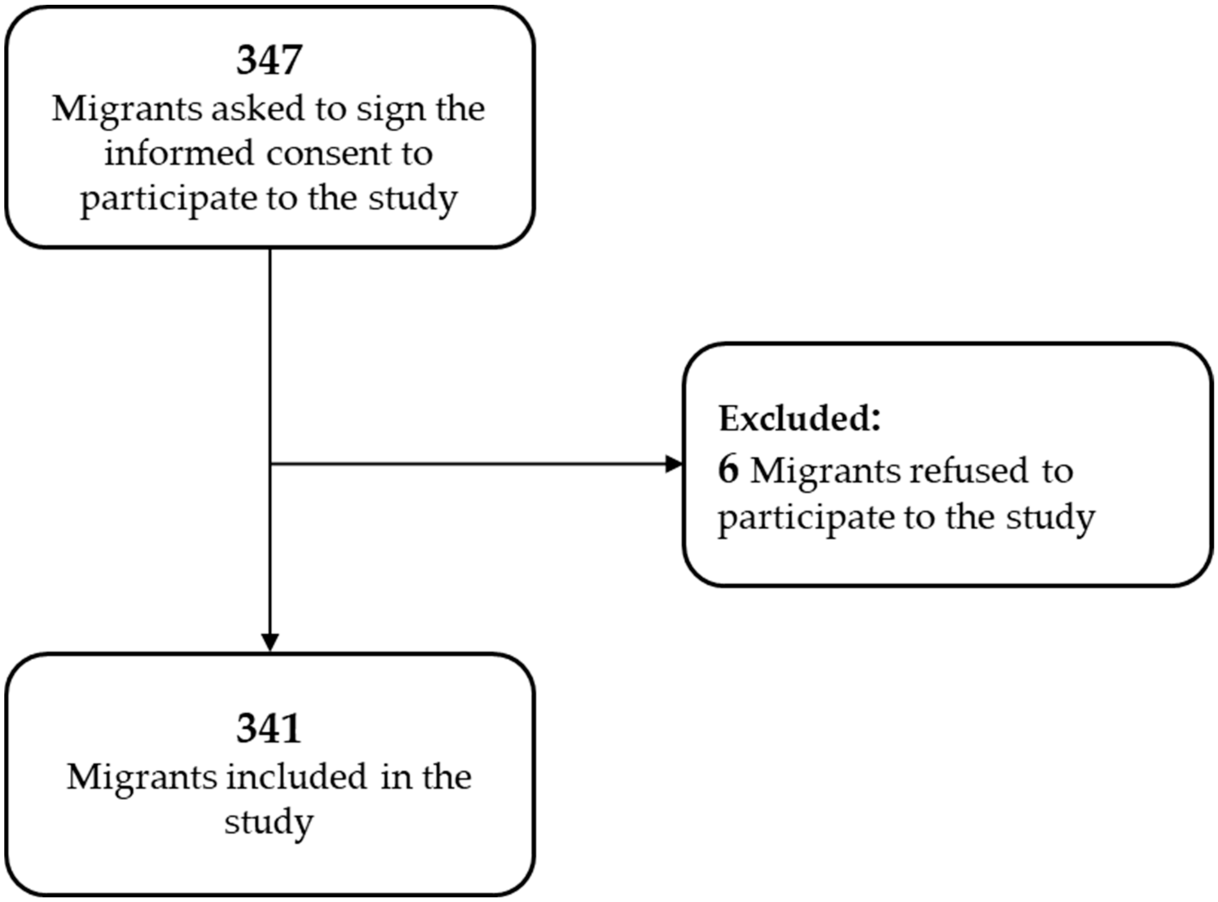

Supplementary Figure S1. Flow-chart of study population selection.

Supplementary Table S1. Prevalence ratio (PR) of being obese or overweight according to individual characteristics. Definition of obesity and overweight based on Body Mass Index. A multiple imputation procedure was adopted to account for missing data.

Total	PR (95% CI) [a]	PR (95% CI) [b]
**Geographic area**		
North Africa	0.75 (0.47–1.18)	0.94 (0.60–1.45)
South Africa	0.56 (0.30–1.05)	0.62 (0.34–1.13)
Latin America	Ref.	Ref.
Asia	0.98 (0.61–1.56)	0.92 (0.58–1.48)
East Europe	0.79 (0.50–1.26)	0.91 (0.60–1.40)
**Sex**		
Male	Ref.	Ref.
Female	0.94 (0.67–1.34)	1.36 (0.97–1.91)
**Age**		
18– 30	Ref.	Ref.
31–40	1.68 (1.00–2.80)	1.10 (0.71–1.71)
41–50	2.11 (1.29–3.45)	1.37 (0.90–2.08)
51+	1.96 (1.17–3.30)	1.36 (0.88–2.11)
**Years spent in Italy**		
≤1	Ref.	Ref.
>1	0.90 (0.65–1.24)	0.76 (0.56–1.03)
**Education***		
Illiterate	1.22 (0.65–2.28)	1.13 (0.62–2.06)
Elementary school	0.93 (0.59–1.47)	0.88 (0.57–1.37)
Middle school	Ref.	Ref.
High school/University	1.01 (0.74–1.38)	1.00 (0.74–1.35)
**Employment***		
Unemployed/Precarious	Ref.	Ref.
Stable	1.13 (0.84–1.52)	1.17 (0.88–1.56)
**House**		
No	Ref.	Ref.
Yes	1.28 (0.95–1.73)	1.10 (0.68–1.76)
**Cohabitants**		
0-1	Ref.	Ref.
2-4	1.17 (0.74–1.86)	1.01 (0.67–1.54)
≥5	1.22 (0.73–2.05)	0.97 (0.60–1.57)
**Adherence to Mediterranean diet**		
Low/Medium	Ref.	Ref.
High	1.31 (0.79–2.17)	1.11 (0.75–1.66)
**Substance use**		
No	Ref.	Ref.
Alcohol and/or Cigarettes	0.91 (0.65–1.28)	1.03 (0.74–1.41)
**Diabetes**		
No	Ref.	Ref.
Yes	1.05 (0.67–1.64)	1.04 (0.66–1.62)
**Hypertension**		
No	Ref.	Ref.
Yes	1.34 (0.90–1.97)	1.23 (0.83–1.80)

[a] Prevalence ratios (PR) of overweigth/obesity based of the Body Mass Index. [b] Prevalence ratios (PR) of overweigth/obesity based of Waist Circumference.
